# Significance of gene therapy in neurodegenerative diseases

**DOI:** 10.3389/fnins.2025.1515255

**Published:** 2025-05-08

**Authors:** Lingling Wang, Lin Ma, Zihan Gao, Ying Wang, Jiaoxue Qiu

**Affiliations:** ^1^Department of Neurology, Yantai Shan Hospital, Yantai, China; ^2^Department of Neurology, Qingdao Municipal Hospital, Qingdao, China; ^3^Department of Internal Medicine of Traditional Chinese Medicine, Affiliated Hospital of Shandong University of Traditional Chinese Medicine, Jinan, China

**Keywords:** gene therapy, Alzheimer's disease (AD), Parkinson's disease (PD), Huntington disease (HD), amyotrophic lateral sclerosis (ALS), spinal muscular atrophy (SMA)

## Abstract

Gene therapy is an approach that employs vectors to deliver genetic material to target cells, aiming to correct genes with pathogenic mutations and modulate one or more genes responsible for disease progression. It holds significant value for clinical applications and offers broad market potential due to the large patient population affected by various conditions. For instance, in 2023, the Food and Drug Administration (FDA) approved 55 new drugs, including five specifically for gene therapy targeting hematologic and rare diseases. Recently, with advancements in understanding the pathogenesis and development of neurodegenerative diseases (NDDs), gene therapy has emerged as a promising avenue for treating Alzheimer's disease (AD), Parkinson's disease (PD), Huntington's disease (HD), amyotrophic lateral sclerosis (ALS), and spinal muscular atrophy (SMA), particularly in personalized medicine. Notably, the FDA has approved three clinical applications for combating SMA, utilizing viral vectors delivered via intravenous and intrathecal injections. However, gene therapy for other NDDs remains in clinical trials, necessitating improvements in viral vectors, exploration of new vectors, optimization of delivery routes, and further investigation into pathogenesis to identify novel targets. This review discusses recent advancements in gene therapy for NDDs, offering insights into developing new therapeutic strategies.

## 1 Introduction

Neurodegenerative diseases (NDDs) have become increasingly prevalent as the global population has aged. Although current therapies for these conditions offer symptomatic relief, they do not alter the progression of the disease, and their effectiveness decreases as the disease progresses (Singh, 2020; Church, 2021). Furthermore, these treatments have limitations: drugs must pass through the blood-brain barrier (BBB) to reach the central nervous system (CNS), which can result in inadequate drug concentrations in the brain. However, for drugs to be effective, they must reach the target tissues; otherwise, they may cause harmful side effects. Gene therapy is a promising approach for delivering genetic material to target cells, allowing the expression of exogenous genes within these cells (Sudhakar and Richardson, 2019). This approach may provide a permanent cure for neurodegenerative diseases by overcoming the limitations of traditional pharmaceutical drug treatment.

NDDs can be divided into genetic and non-genetic categories, which often have an insidious onset and slow progression, affect individuals across all age groups, and impose significant financial burden on patients' families and society. According to relevant studies conducted between 2019 and 2022 at home and abroad, the following findings have been reported: Parkinson's disease (PD) affects ~1.37% of the population aged > 60 years, and the number of PD patients in the elderly population is anticipated to triple worldwide from 1990 to 2016 (GBD 2016 Neurology Collaborators, 2019; Qi et al., 2021). The incidence of Huntington's disease (HD) in the Americas, Oceania, and Europe is increasing annually, with evidence indicating a 15–20% increase every 10 years (Rawlins et al., 2016). Alzheimer's disease (AD) affects 3.94% of all individuals over 60 years of age and has a global prevalence of 50 million individuals (Jia et al., 2020). Amyotrophic lateral sclerosis (ALS) and spinal muscular atrophy (SMA) are rare diseases with high mortality rates, and their annual incidence is increasing worldwide (Feldman et al., 2022; Mercuri et al., 2022). Additionally, AD has resulted in substantial economic losses in China and other regions globally, with the global cost of dementia estimated to reach $9.12 trillion by 2050 (Jia et al., 2018).

NDDs have complex mechanisms, and their precise pathogenesis remains unclear. The common pathological feature of NDDs is the aggregation of misfolded proteins in specific tissues of the CNS, which induces the dysfunction and death of various types of neurons, ultimately leading to deterioration of brain function (Vaquer-Alicea and Diamond, 2019). Gene therapy has been designed as an efficient strategy to counteract the pathological features of NDDs. Gene therapy involves several approaches, including the widespread silencing of misfolded proteins, expression of therapeutic proteins and antibodies, and restoration of normal gene editing of misfolded proteins to achieve successful protein expression (Paunovska et al., 2022; Tang and Xu, 2020). However, these strategies have not achieved remarkable results in early phase clinical trials. Currently, major efforts are focused on increasing the effectiveness of gene therapies by identifying new vectors, novel therapeutic targets, and reliable delivery routes for transgenes (Chen et al., 2020). Substantial progress has been made in gene therapy for NDDs. Herein, we review these novel findings to provide a reference for both basic and clinical research.

## 2 Potential therapeutic strategies of gene therapy in NDDs

Human gene therapy has emerged as a promising treatment for acquired nervous system disorders, which can deliver complementary DNA to the pathological region of the CNS to realize the expression of therapeutic proteins and partially restore neuronal function (Simonato et al., 2013). In phase I clinical trials of AD, stable expression of nerve growth factor (NGF) in the patient's brain was successfully achieved through bilateral stereotactic injections into the nucleus basalis of Meynert in the forebrain of AD (Hudry and Vandenberghe, 2019; Rafii et al., 2014). Antisense oligonucleotide (ASOs), miRNAs (microRNAs), shRNAs (short hairpin RNAs), clustered regularly interspaced short palindromic repeats (CRISPR)/Cas9 and siRNAs (small interfering RNAs) are designed to realize the effect on the pathogenic precursor mRNA (pre-message RNA, pre-mRNA) and mRNA levels to silence its expression. ASOs are a promising alternative therapeutic platform given high specificity and variety of chemical modification. Currently, nine therapeutic ASOs are approved by the FDA, which have been used in the treatment of rare diseases and show favorable safety profile and encouraging efficacy (Crooke et al., 2021). In clinical trials of HD, ASOs (IONIS-HTTRx) targeting the silencing of mutated huntingtin protein (mHTT) effectively reduced mHTT protein expression (Tabrizi et al., 2019). As miRNAs are endogenous components, the re-introduction of these miRNAs into cells may be well tolerated. Although many studies have been done with miRNA, none has resulted in approval for medical use by the FDA (Yu et al., 2020). In AD mouse models, miRNAs (miRCD33) designed to silence siglec-3 (CD33) expression effectively inhibited the deposition of β-like amylase (amyloid-β; Griciuc et al., 2020). ShRNA is composed of 19–20 base pair RNA sequence containing a short hairpin loop, which has been introduced for highly specific gene silencing. Due to the susceptibility to degradation and off-target effects, delivering shRNA into target cells is partly under clinical trial and face many challenges in practice (Acharya, 2019). In PD rat models, shRNA targeting silencing of Rho-associated kinase (ROCK) prolonged the survival of dopaminergic neurons (Saal et al., 2015). CRISPR/Cas9 is able to generate DNA double-strand breaks with specificity for targeted sites, which is used to regulate mutation site correction, epigenetic modification, transcriptional regulation, and gene expression silencing of disease-causing genes at the DNA and mRNA levels (Pacesa et al., 2024). In mouse models of AD, targeted silencing of beta-secretase 1 (BACE1) using CRISPR/Cas9 technology can effectively inhibit the formation of amyloid-β aggregates (Park et al., 2019). However, CRISPR/Cas suffer from enzymatic degradation in the physiological environment, off-target activity and low permeability into cells (Lin et al., 2014). Nowadays, there are three types of cargo formats to protect the CRISPR system from degradation and enhance their tissue targeting, that is, Cas: single-guide RNA ribonucleoprotein, Cas mRNA and single-guide RNA, and Cas plasmid expressing CRISPR/Cas systems (Xu et al., 2022). Despite the great therapeutic potential of these RNA molecules, a number of critical issues still need to be addressed, such as stability, targeting specificity and the efficiency of delivery (Yu et al., 2020). Whether the exogenous therapeutic substances mentioned above can exert the expected efficacy in the clinical treatment of NDDS depends on the choice of delivery vehicle and route.

## 3 Vectors and delivery routes of gene therapy in NDDs

The efficacy of gene therapy is greatly influenced by delivery vectors, which can be broadly classified into two categories: viral and non-viral vectors. Viral vectors are divided into five distinct types: adenoviruses (Advs), Lentiviruses (LVs), Retroviral vectors (RVs) and herpesviral vectors (HSV), and adeno-associated viruses (AAVs). Adenovirus (Advs) vectors can serve as an efficient vector for gene therapy due to high-efficiency transduction and low pathogenic activity. In addition, Adv have relatively transient expression and high immunogenicity, which have be mainly used in the prevention of infectious diseases and treatment of cancer (Barkats et al., 1998). LVs vectors belong to the Retroviridae family, which can provide a long-term and stable gene expression can and relatively low immunogenicity. Currently, LVs vectors have mainly been used for *ex vivo* gene therapy applications (Li et al., 2023). RVs is an enveloped spherical virus which can harbor a large gene and lead to long-term gene expression. However, RVs vectors undergo random integration into host chromosomal DNA, and are not frequently used in clinical studies (Yi et al., 2005). HSV is an enveloped virus which can evade the immune system and deliver large DNA cargos. Given its intrinsic oncolytic capability, HSV vectors have been developed for cancer therapy (Manservigi et al., 2010). AAVs is a non-pathogenic parvovirus and do not integrate DNA into host genome and enable long-term stable gene expression. In summary, each viral vector possesses unique characteristics and can be used for various purposes. The development of recombinant AAV vectors provided more options for various diseases owing to its advantages, including tissue tropism, specificity in transduction, eliciting a relatively low immune response, no incorporation into the host chromosome, and persistent foreign gene expression (Wang et al., 2024). Currently, AAVs vectors have been most widely applied in clinical trials and basic research of NDDs (Ling et al., 2023). 13 AAV serotypes and more than 100 variants have been identified and classified (Pupo et al., 2022). Among them, adenovector AAV2 is the preferred delivery vector for PD, as it is relatively safe and introduces exogenous genes into the chosen regions of the CNS to persist (LeWitt et al., 2011; Marks et al., 2010, 2008). AAV11 exhibits powerful and highly specific retrograde labeling of projecting neurons in multiple neural circuits in mouse models. Additionally, AAV11 plays a crucial role in monitoring neuronal activity in functional networks, investigating neuron-astrocyte connections, and analyzing differences in circuit connectivity. These properties make AAV11 a promising candidate for NDDS (Han et al., 2023). Additionally, reports have highlighted potential biosafety concerns related to AAV vectors, with AAV-RNAi vector delivery causing cerebellar toxicity in the brains of non-human primates. Despite these potential biosafety risks, it is expected that advancements in clinical research will address and mitigate issues related to adverse effects and therapeutic efficacy (Keiser et al., 2021).

Non-viral vectors transport foreign genetic material into cells based on the physicochemical properties of the synthetic materials. Unlike viral vectors, non-viral vectors have a number of advantages, such as larger packaging capacity, ease of production, lower immunogenicity and high safety (Sainz-Ramos et al., 2021). The efficient non-viral vectors mainly include liposomes and lipid nanoparticles (LNPs), cationic polymers, exosomes, inorganic nanoparticles and polymer hydrogels. Among them, LNPs and cationic polymeric based vehicles have emerged as perhaps the most promising non-viral gene vector candidates, which have the advantages of powerful gene loading capacity, superior safety and practicality as well as simplicity of preparation (Wang et al., 2023). Currently, two types of mRNA vaccines with LNP delivery vehicles have been approved on the market. LNPs gained a promising prospect as a carrier due to their high biocompatibility and biodegradability. However, achieving specific RNA delivery to extrahepatic tissues and the immunogenicity of LNPs become the limiting factor (Ma et al., 2024). Cationic polymers have many positively charged groups in the molecules, which are capable of forming nanoparticles with nucleic acids through electrostatic interactions (Pack et al., 2005). Cationic polymers have been explored to carry plasmid DNA, mRNA, and siRNA for gene delivery, with achievement of therapeutic functions, such as gene augmentation, gene suppression, and genome editing. Due to easy synthesis and multiple modifications, cationic polymer-based gene therapy offer great potential in pre-clinical phases, but the effect on the stability of cellular membranes must remain an important factor for consideration in treatment decision-making (Cai et al., 2023).

In recent years, tremendous efforts have been made to develop Biomimetic nanovesicles (BNVs) as novel non-viral delivery systems. BNVs are divided into endogenous extracellular vesicles (EVs) and composite artificial nanovesicles (ANVs). EVs are derived from various kinds of cells including exosomes, microvesicles and apoptotic bodies (ApoBDs). As a naturally occurring vector for gene delivery, EVs have been widely used for siRNAs and miRNAs. Moreover, EVs has enormous potential to deliver mRNAs, CRISPR/Cas systems and ASOs. An increasing number of studies have exhibited many strengths of EVs, such as high biocompatibility, low immunogenicity, intrinsic targeting capacity, high cycling stability and BBB hyperpermeability, However, its clinical translation is constrained by large-scale and standardized production and an effective strategy for loading nucleic acids into EVs. Currently, several clinical trials involving EVs are in progress (Lu et al., 2023; Gargiulo et al., 2020). ANVs, synthesized by artificial nanotechnology, are designed to overcome this problem. ANVs present many advantages, such as low cost, huge production yields, multifunctionality, biosafety and immune escape (You et al., 2024). Currently, combining BNVs with various nanomaterials has been widely used to treat NDDs due to these properties. Researchers have developed a biomimetic nanozyme (CuxO@EM-K) with amyloid-β-targeting pentapeptide KLVFF, which lead to a reduction of Aβ levels in blood and brain tissues and ameliorated memory deficits without apparent toxic effects (Ma et al., 2020). REXO-C/ANP/siRNA targeting SNCA can act as a nanoscavenger, which could promote clearance of α-synuclein aggregates and improve motor symptoms in a PD mouse model (Liu et al., 2020a). The characteristics of the viral and non-viral vectors are summarized in Table 1.

**Table 1:** Comparioson of viral vectors and non-viral vectors in gene therapy.

**Vector type**	**Advantages**	**Disadvantages**
Viral vectors	1. Relative stable transgene expression; 2. Spatial and temporal expression of gene; 3. Specific vector	1. Lower production efficiency; 2. Susceptible to immune reactions; 3. The maximal packaging capacity r is 4.5 kb 4. Harder safety assessments 5. Genotoxic properties.
Non-viral vectors	1. Easy production 2. Lower costs 3. Less susceptible to induce an immune response. 4. Larger packaging capacity	1. lower transfection efficiency 2. Lower intrinsic toxicity. 3. Lower specificity to cells. 4. High doses requirment

**Figure 1:**
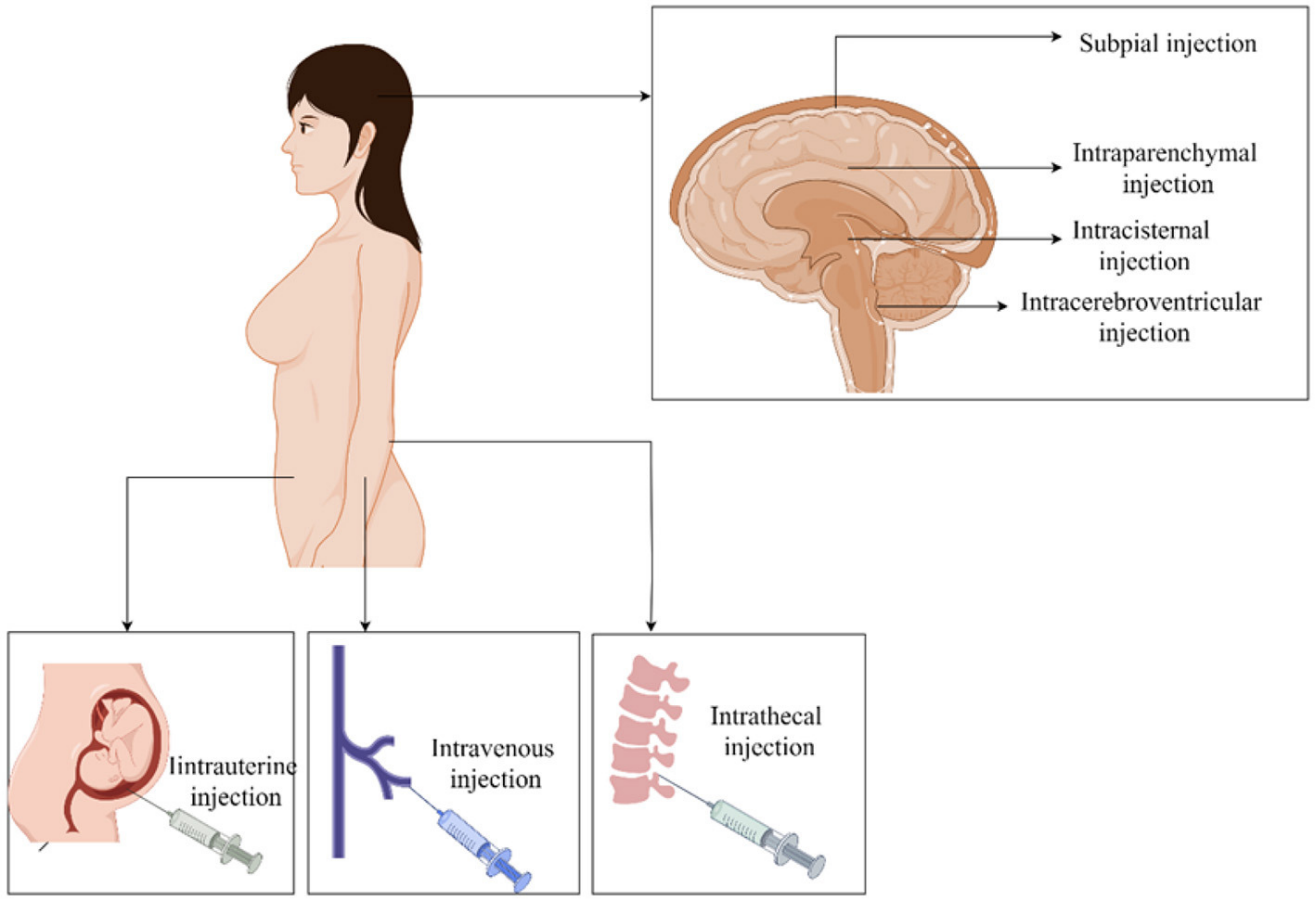
Delivery routes of gene therapy in neurodegenerative disease. Although intrave nous or central nervous system delivery (intrathecal, intracerebroventricular, intracisternal and subpial routes) administration can effectively treat multifocal disorders, intraparenchymal injection is the most frequently applied delivery route for brain diseases. Intrauterine injection is used to treat inherited NDDs.

**Figure 2:**
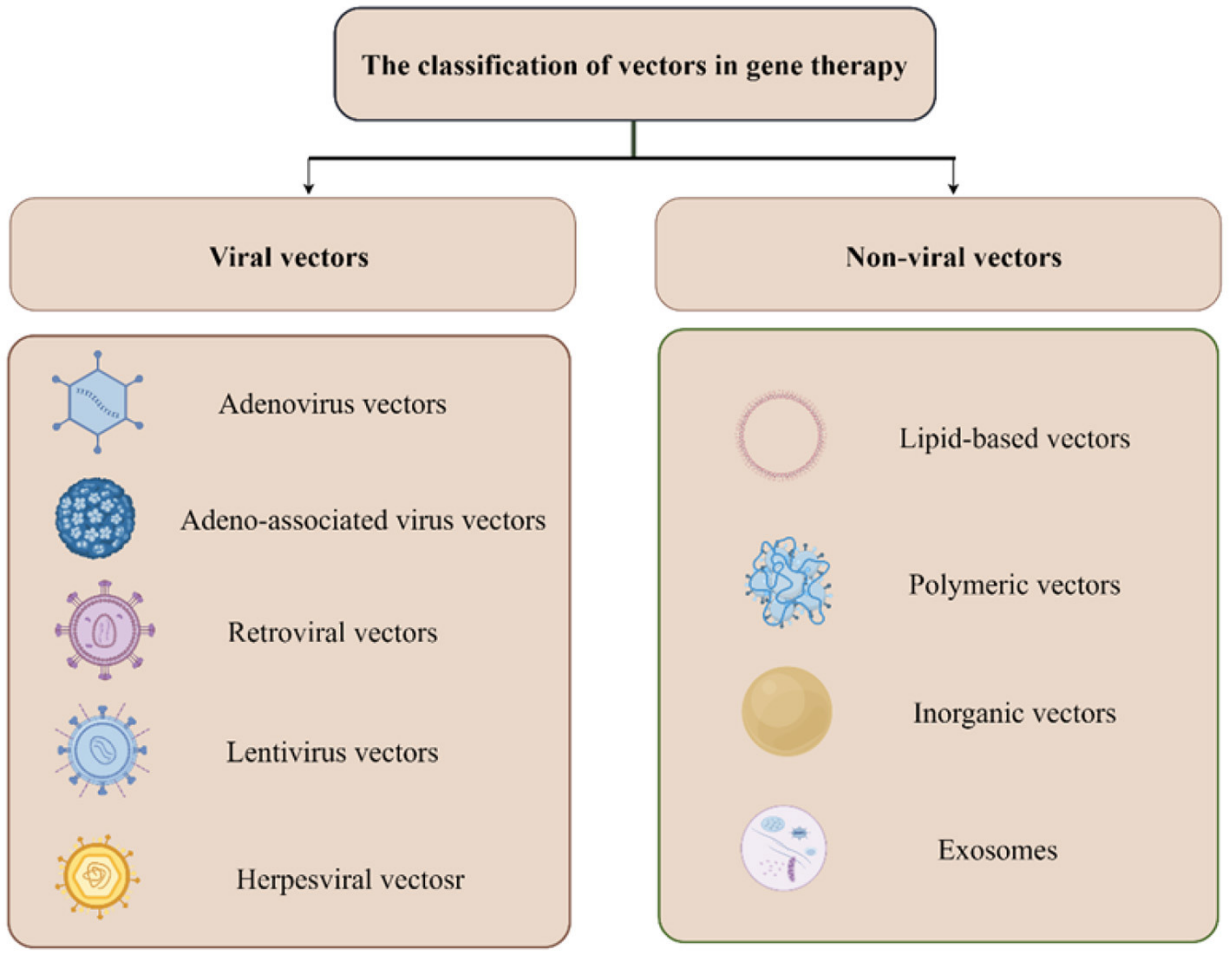
Introduction of Viral and non-viral vectors.

Gene therapies have been developed and are currently being employed in clinical trials and the treatment of neuromuscular diseases (NMDs). It is essential to choose appropriate delivery routes or injection methods as they determine the effectiveness of vectors for different diseases. Intravenous injections can deliver vectors intravenously to exert their therapeutic effect on the CNS. However, the therapeutic effect of this approach is limited because of restricted access to CNS. Therefore, not all vectors are suitable for this delivery method, with the exception of AAV9 and retrofitted AAV9 vectors, such as AAV-B1 and AAV-AS (Choudhury et al., 2016a,b; Mattar et al., 2013). Intraparenchymal injection involves the direct injection of the vector into pathological brain tissue to achieve therapeutic purposes. Considering its high invasiveness, intraparenchymal injection was used only once in a lifetime (Gao et al., 2024). Viral vectors, such as AAV1, AAV2, AAV6, AAV8, and AAV9, and non-viral vectors are suitable for this delivery method (Cearley and Wolfe, 2007). Intrauterine injections could be a potential therapeutic option for inherited neurodegenerative diseases. Integration-deficient lentiviral vectors (IDLVs) enable efficient and permanent transduction of motor neurons (MNs) after intrauterine injection, suggesting that IDLVs may be efficient tools for the treatment of inherited neurodegenerative diseases (Ahmed et al., 2018). CNS delivery directly delivers the vector into various tissues and organs of the CNS, unaffected by the blood–brain barrier. The different injection sites can be divided into intrathecal, subpial, intracisternal, and intracerebroventricular injection (Levites et al., 2006; Miyanohara et al., 2016; Samaranch et al., 2012). Although this route of administration can convey drugs to widely distributed brain regions, rapid clearance from the CSF and multiple repeated injections decrease patient compliance (Naseri Kouzehgarani et al., 2021; Soderquist and Mahoney, 2010). Non-viral vectors and viral vectors such as rAAV9 and rAAVrh.10 are a good fit for this delivery route (Gao et al., 2024; Słyk et al., 2024). In addition to delivery routes, choosing cell-type specific promoters is critical for achieving expected therapeutic effects. Some studies have found that the synapsin-1 (Syn1) and the neuron-specific enolase (NSE) promoter can specifically label neurons to achieve treatment goal without evoking any off-target effects (Kügler et al., 2001). We illustrated the delivery routes of gene therapy in NDDs in Figure 1. We also demonstrated the viral and non-viral vectors in Figure 2.

Beyond viral and non-viral delivery vectors, a small molecule-regulated system for modulating gene therapy through drug-induced splicing has been reported. This system enables precise control of gene replacement or editing upon exposure to small molecules, thus overcoming the limitations of traditional gene therapies in precisely controlling complex genetic structures. This system may represent another promising research avenue with substantial developmental potential beyond current delivery vectors, and its integration with existing clinical therapeutics could expedite clinical advancements (Monteys et al., 2021).

## 4 Gene therapy in AD

Alzheimer's disease (AD) is a neurodegenerative disorder that exhibits unique pathological features, including accumulation of β-amyloid deposits and tau tangles. The primary symptom of AD is progressive cognitive impairment, with memory loss often the first sign. As the disease progresses, patients experience increasing personality and behavioral changes, and in the late stages of the disease, they require extensive support from friends and family caregivers (Scheltens et al., 2021). Despite the numerous hypotheses proposed to explain the pathogenesis of AD, such as the amyloid cascade hypothesis, Tau hypotheses, mitochondrial metabolic reprogramming of ApoE, oxidative stress hypothesis, calcium homeostasis hypothesis, neurovascular hypothesis, metal ion hypothesis, and lymphatic system hypothesis, the mechanistic basis for AD remains unclear. Currently, existing drug treatments are ineffective in addressing the complex pathogenesis of AD and there is a need for more efficacious treatments. Advances in gene therapy have shed light on the experimental treatment of AD, and molecules, such as NGF, MAPT, APP, TRIM11, TREM2 and apoE, which play critical roles in the onset and progression of AD, may serve as promising therapeutic targets (Nagahara et al., 2009; Scheltens et al., 2021; Strang et al., 2019; Zhang et al., 2023).

Nerve Growth Factor (NGF) is an endogenous neurotrophic factor that regulates the survival and growth of cholinergic neurons in the basal forebrain. An increase in NGF has been shown to correlate with an increase in the activity of choline acetyltransferase (ChaT) in the basal forebrain, and this effect is dose- and region-specific (Mobley et al., 1986). A decrease in NGF expression in cholinergic neurons can lead to cell senescence and death, resulting in a decline in cognitive function, suggesting that NGF plays a significant role in the progression of Alzheimer's disease (AD). Furthermore, the injection of cDNA transduced with AAV2 encoding NGF into cholinergic neurons can promote stable, long-term expression and neuronal survival (Nagahara et al., 2009). However, despite these promising results, the role of NGF remains limited and its ability to restore cognitive function in patients with AD has not yet been demonstrated (Rafii et al., 2018).

Tau is a microtubule-associated protein encoded by the MAPT gene, and mutations in MAPT lead to the over-phosphorylation and aggregation of the Tau protein, which in turn leads to the occurrence of neurofibrillary tangles in the CNS, which is the initial pathological manifestation of AD and positively correlates with cognitive impairment. The use of ASOs to degrade the pre-mRNA of MAPT (ASOs-MAPT) can effectively inhibit the expression of mutant tau proteins in the animal and cell models (DeVos et al., 2017; Sud et al., 2014). Subsequently, a phase 1b clinical trial of a MAPT-targeting ASO confirmed these findings, which showed that mild AD patients have reduced tau in the cerebrospinal fluid (CSF; Mummery et al., 2021). Owing to off-target effects of ASO and difficulty in passing through the blood-brain barrier, these current studies open new hopes in translating ASO into the clinic (Scoles and Pulst, 2018).

Amyloid-β is widely found in all types of neuronal synapses, which is formed by the processing of amyloid precursor protein (APP). Mutations in the gene encoding APP protein led to the formation of oligomers of processed amyloid-β, followed by aggregation, which is positively associated with the development of AD. Recent gene therapy-based studies have shown that AAV vectors can be utilized to piggyback on CRISPR/Cas9 technology to form components that silence the allele (allele) where the APP mutation occurs without affecting the expression of the WT-APP gene on the other allele (György et al., 2018). Using NHEJ-based CRISPR/Cas9 technology to mutate exon 18 of APP, this mutation may cause an APP conformational change, which may prevent its degradation of amyloid-β (Sun et al., 2019). The γ-secretase cleavage site encoded by exon 17 of the APP gene controls the cell membrane localization of the APP protein as well as the secretion of amyloid-β into the extracellular compartment, and ASOs targeting exon-17 pre-mRNA (ASOs-exon17) can effectively reduce the formation and secretion of amyloid-β (Chang et al., 2018). Recent studies have shown that some proteins in the brain are utilized to remove amyloid-β or to inhibit its function. Low-density lipoprotein receptor-related protein 1 (LRP1) is a receptor for amyloid-β on endothelial cells, and when LRP1 binds amyloid-β, it transports amyloid-β to lysosomes for degradation, with expression levels inversely correlating with the development of AD (Herz et al., 1988). Using the AAV2 vector containing cDNA from the ankyrin repeat and SAM domain containing 1 A (ANKS1A) gene, the expression of the ANKS1A protein in endothelial cells can activate LRP1 and promote the formation of a complex between LRP1 and amyloid-β to be transported to the lysosome to remove amyloid-β (Lee et al., 2023). AAV vectors can also be used to express the antibody NUsc1, specifically targeting amyloid-β in the CNS, which can effectively inhibit the binding of extracellular amyloid-β to neurons (Selles et al., 2023).

Tripartite motif protein 11(TRIM11), acting as a molecular chaperone for tau, has strong activity to clear tau aggregates and is significantly down-regulated in brains of individuals with AD. TRIM11 was silenced with several ASOs against TRIM11(ASOs-TRIM11) in cultured cortical neurons, which can obviously increase AT8- and MC1-reactive tau and decrease presynaptic marker synaptophysin (SYP) positive puncta and postsynaptic density protein 95 (PSD95) positive puncta. Conversely, cortical neurons transduced with AAV9 encoding TRIM11 (AAV9-TRIM11) makes the expression of TRIM11 upregulated, thereby significantly reducing AT8- and MC1-reactive Tau and increasing SYP- and PSD95-positive puncta. These results indicate TRIM11 can prevent the accumulation of tau, provide neuronal protection and maintain the viability of the neurons (Zhang et al., 2023).

Triggering receptor expressed on myeloid cells 2 (TREM2), a transmembrane glycoprotein, is one of the most highly expressed receptors in microglia and has been widely considered to have an important role in AD pathogenesis (Qin et al., 2021). The overexpression of TREM2 correlates with some improvements in microglial endocytosis of Aβ, making TREM2 a possible therapeutically target for AD treatment. However, poor penetration of the BBB and non-selective drug distribution in specific brain regions limits its clinical application. The biomimetic nanovesicles named TSEL can facilitate the drug aggregation at AD lesions, which contains β-secretase BACE1 siRNA (siBACE1) and TREM2 plasmid (pTREM2). Knockdown of BACE1 via siRNA resulted in reduced amyloid plaques and improved cognitive functions. Meanwhile, the up-regulation of TREM2 can modify microglia phenotypes and restore its capacity to phagocytose Aβ and promote the neurorestorative function. The results have shown that biomimetic nanovesicles can deliver dual nucleic acids to realize the synergistic gene therapy for AD, providing a new strategy for developing novel medicines for treating AD (Jiang et al., 2024).

Apolipoprotein E (apoE) is a multifunctional protein that serves as a lipid carrier, which plays a central role in lipid metabolism and the maintenance of neuronal homeostasis in both the peripheral and central nervous systems. It exists in three major isoforms: apoE2, apoE3, and apoE4, each of which exerts distinct effects on the repair of neuronal injuries (Huang and Mahley, 2014; Zhao et al., 2018). Among them, E2 has a neuronal protective function, and the expression of E4, which can interact with amyloid-β to cause neuronal synaptic dysfunction and neuroinflammation, is positively correlated with the onset of Alzheimer's disease, but negatively correlated with age (Jackson et al., 2024). E4 (Arg112), the amyloid-β binding site, differs from E3 (Cys112) only by one amino acid residue (Yu et al., 2014). Conversion of apoE4 to apoE3 by using CRISPR/Cas9 genome editing can alleviate AD related pathologies in induced pluripotent stem cells (iPSCs; Lin et al., 2018). The delivery of E2 cDNA (AAV-E2) via an AAV vector can elevate the expression of E2, which can also preserve neurons and avoid amyloid-β deposition (Hudry et al., 2013). However, the pathogenesis of AD has not been elucidated. An appreciation of the pathogenesis of AD is therefore essential for the treatment of AD using gene therapy.

## 5 Gene therapy in PD

Parkinson's disease (PD) is a multisystem neurodegenerative disorder with a complex pathogenesis characterized by the death of dopaminergic neurons in the substantia nigra pars compacta (SNc) of the CNS, followed by dysregulation of dopamine secretion and disruption of nigrostriatal projections (Obeso et al., 2008). Additionally, disruption of nigrostriatal projections can lead to abnormal regulation of the basal ganglia network, which in turn induces resting tremors, muscular rigidity, hypokinesia, and impaired balance (Grayson, 2016). To improve PD associated symptoms, conventional drug therapy can only partially improve the clinical symptoms of PD and induce significant side effects. Currently, the therapeutic targets, such as SNCA, GBA1, LRRK2, GDNF and NRTN, have been reported to participate in the development of PD and may offer an alternative pharmacological treatment for PD.

Mutations in the distal enhancer of SNCA, the gene encoding α-synuclein, lead to aberrant ex pression and oligomerization of α-synuclein, which then induces the formation of α-synuclein aggregates in dopaminergic neurons, ultimately leading to the death of dopaminergic neurons and the development of PD (Soldner et al., 2016). Recent studies have shown that a complex composed of CRISPR-deactivated Cas9 (dCas9) technology-associated constitutive elements and DNA-methyltransferase 3A (DNMT3A) using AAV vectors promotes the methylation of mutant SNCAs, which can reduce the formation of oligomeric α-synuclein (Kantor et al., 2018). Similarly, the vector system is fused with a synthetic repressor molecule comprising the transcription repression domain (TRD) of the DNA methyltransferase Krüppel-associated box (KRAB)/methyl CpG binding protein 2 (MeCp2), and then MeCp2-TRD binds to dCas9 to inhibit SNCA transcription, thereby repressing SNCA transcription with higher efficiency (Sun et al., 2024). In addition, mRNA reduction triggered by ASOs or shRNA (ASOs-α-synuclein/shRNA-α-synuclein) inhibits the translation of mutant α-synuclein (Zharikov et al., 2015; Uehara et al., 2019). Gold nanoparticle composites (NPs) loaded with plasmid DNA (pDNA) was found to significantly increase the tyrosine hydroxylase (TH) level and reduce formation of α-syn aggregates, thereby improving motor dysfunction and reducing exploration capacity in PD mice (Liu et al., 2020b). USP30, a deubiquitinase that targets mitochondrial proteins, inhibits mitochondrial autophagy (mitophagy) to remove pathological proteins. Given this property, improved mitochondrial function was obtained upon USP30 depletion in PD, which could efficiently clear α-synuclein and prolong the growth cycle of dopaminergic neurons. Thereafter, gene therapy strategies such as the CRISPR/Cas9 system, RNAi, and ASOs could be utilized to silence USP30 expression for PD treatment (Fang et al., 2023).

Mutations in the GBA1 gene encoding lysosomal glucocerebrosidase (GCase) cause reduced ex pression of GCase, leading to the inability to degrade protein aggregates within the lysosome, suggesting that the expression of GCase is negatively associated with the development of PD (Do et al., 2019). AAV vectors are used to deliver GCase (AAV-GBA1) to unilateral striatum to enhance the expression of GCase in transgenic A53T α-synuclein mice, which in turn leads to a significant reduction in soluble α-synucleinin (Sardi et al., 2013). In addition, an allosteric activator of GCase, LTI-291, can effectively activate the expression of GCase in PD patients, but there was no alteration in UPDRS before and after treatment (De Heijer, 2023). Thus, GCase activity could be critical in preventing α-syn accumulation in PD, demonstrating that the development of carrier-mediated molecules targeting GCase expression or activity could provide a novel focus for PD therapeutics through a potential mechanism.

Pathological studies of Parkinson's disease (PD) have shown that pathogenic mutations in the leucine-rich repeat kinase 2 (LRRK2) gene result in increased LRRK2 kinase activity, thereby promoting disease progression. Small molecule inhibitors targeting LRRK2 kinase have demonstrated neuroprotective effects in both preclinical and clinical translational studies of PD, making them a promising therapeutic strategy for the disease (Kingwell, 2022). The development of drugs targeting kinase activity is a major current focus, with two LRRK2 kinase inhibitors, DNL201 and DNL151, undergoing clinical development. Additionally, other inhibitors, such as MLi-2 and PF-06685360, are currently in active preclinical development (Tolosa et al., 2020; Jennings et al., 2022). An alternative approach utilizes antisense oligonucleotides (ASOs) to reduce LRRK2 expression levels, thereby blocking LRRK2 activity independently of specific mutations and their functional consequences. In PD mouse models, intracerebroventricular administration of LRRK2-targeted ASOs has been shown to decrease LRRK2 protein levels, reduce α-synuclein inclusions, and attenuate the degeneration of dopaminergic neurons in the substantia nigra (Zhao et al., 2017). However, due to the tissue-specific expression of LRRK2, inhibitors targeting LRRK2 may not always be protective; for example, the reduction of LRRK2 levels could lead to pulmonary fibrosis (Hu et al., 2023; Tian et al., 2021). Therefore, the safety assessment of LRRK2-targeted inhibitors, along with tissue-specific delivery and dosing, are likely to become key areas of focus in future research.

The glial cell line-derived neurotrophic factor (GDNF) family of neurotrophic factors that regulate the survival and function of neurons. Neurturin (NRTN), a homologous neurotrophic factor to GDNF, plays a crucial role in protecting nigral dopaminergic neurons and alleviating behavioral symptoms in animal models of PD (Bartus et al., 2011; Gasmi et al., 2007). Gene therapies targeting GDNF and NRTN have also shown promising results. Both GDNF and NRTN can enhance the survival rate of dopaminergic neurons (Tomac et al., 1995; Peterson and Nutt, 2008; Collier and Sortwell, 1999). The delivery of GDNF-expressing cDNA into dopaminergic neurons using the AAV2 vector (AAV2-GDNF) leads to GDNF expression within these neurons and prolongs their survival (Kells et al., 2004; Ciesielska et al., 2011; Su et al., 2009). The receptor RET tyrosine kinase, serving as the classical receptor for both GDNF and NRTN, plays a critical role in promoting the development and protection of dopaminergic neurons in PD models through tandem or independent signaling (Drinkut et al., 2016). GDNF can prevent the accumulation of misfolded α-synuclein in dopaminergic (DA) neurons, and constitutively active RET can protect DA neurons by reducing fibril-induced α-synuclein accumulation. These therapeutic effects on disease phenotypes disappear with the loss of RET or the use of related inhibitors (Drinkut et al., 2016; Chmielarz et al., 2020). The targeted use of small peptide molecules and specific RET agonists has proven effective in protecting dopaminergic neurons and treating PD in animal models (Renko et al., 2021; Mahato and Sidorova, 2020).

GABA plays a protective role in dopaminergic neurons, which is synthesized from glutamate by glutamate decarboxylase (GAD). Studies have shown that delivery of GAD-expressible cDNA using the AAV2 vector (AAV2-GAD) results in stable expression of GAD in the brains of patients with PD, thereby significantly improving motor symptoms (LeWitt et al., 2011; Kaplitt et al., 2007). Meanwhile, activation of striatal neurons may represent a useful strategy to alleviate PD motor symptoms. The retrograde AAV vector-targeted D1 MSNs which contains potent D1-MSN-specific promoters, and a chemo-genetic effector are designed to activate the D1 MSNs, which play a role in ameliorating motor disabilities (Chen et al., 2023). The researchers have designed and synthesized transgenic tool capable of retrograde labeling striatum SPNs, which consists of retrograde AAV8R12 with new promoters, thereby reversing the core motor deficits in PD animal models (Yan and Jin, 2024). In addition to protection against neuronal loss, replacement of lost neurons is also an option. ASOs bind to target polypyrimidine tract-binding proteins (PTBs), one of the most investigated RNA-binding proteins (RBP), and silence PTB expression, which promotes the conversion of dormant astrocytes into dopaminergic neurons and shows beneficial effects in PD models (Yang et al., 2023; Qian et al., 2020).

The dopamine synthesis pathway (DSP) is controlled by three key enzymes: tyrosine hydroxylase (TH), aromatic amino acid decarboxylase (AADC), and GTP-cyclohydrolase 1 (GCH1; Shen et al., 2000; Muramatsu et al., 2002; Sun and Roy, 2021). AADC catalyzes dopamine formation. Downregulation of AADC expression is correlated with the development of PD. Delivery of AADC-expressible cDNA (AAV2-cDNA) using the AAV2 vector can achieve sustained expression of AADC in dopaminergic neurons and promote dopamine synthesis to ameliorate some of the patients' symptoms (Christine et al., 2022; Hadaczek et al., 2010; Christine et al., 2009; Mittermeyer et al., 2012). PD is a neurodegenerative disease with a complex pathogenesis, and it is necessary to accurately analyze the major cause of morbidity in patients with PD and use a combination of gene therapies targeting multiple causative factors to achieve the goal of curing the disease.

## 6 Gene therapy in HD

Huntington's disease (HD) is an autosomal dominantly inherited neurodegenerative disease caused by trinucleotide CAG repeat expansion in exon-1 of the Huntington's gene (huntingtin, HTT; MacDonald et al., 1993). Mutant HTT expresses polyglutaminated huntingtin (mHTT) protein, which enhances aggregate formation in MSNs and cortical pyramidal neurons (CPNs). HD occurs in people around 40 years of age, and its early onset is characterized by involuntary movements accompanied by gait disturbances. As the disease progresses, patients clinically present with motor, mental, and cognitive dysfunction (Ross and Tabrizi, 2011). Some studies have found that small molecule targeted therapies functions by promoting the inclusion of a pseudoexon in the primary transcript, which facilitates brain penetration upon oral administration, leading to a reduction in mHTT protein levels (Keller et al., 2022). Currently, clinical treatments for HD are limited to the use of drugs that degrade mutant Huntington's proteins, thereby delaying disease progression (Yamamoto et al., 2000; Tabrizi et al., 2022). Gene therapy can silence or correct the expression of mHTT proteins at the genetic level to cure disease. AAV2 vectors are delivery miRNAs (AAV2-miHTT) and shRNAs (AAV2-shHTT) targeting the silencing of HTT protein expression, which reduces the number of mHTT proteins by ~45%, resulting in the partial alleviation of disease symptoms (McBride et al., 2011; Grondin et al., 2012). Further studies have shown that AAV5 vectors carrying modified transgene-engineered miRNAs can reduce the number of mHTT proteins by more than 80% and achieve improved therapeutic effects (Evers et al., 2018). AAV vectors can also be utilized to reduce the expression of the mHTT protein by carrying CRISPR-Cas13d technology-related components targeted at silencing HTT protein expression (Morelli et al., 2023).

The above gene therapy strategies applied to clinical treatment are highly generalizable, even if individual patients have specific single nucleotide polymorphisms (SNP), but they do not affect the therapeutic effect. This shortcoming is that the expression of the wild-type (WT) HTT protein is also inhibited, which affects its regulatory role in the organism, leading to acute pancreatitis, homeostatic imbalance in the brain, and other complications (Wang et al., 2016; Dietrich et al., 2017). Therefore, selective silencing of the mHTT protein without affecting the expression of the WT-HTT protein has been the development trend of clinical gene therapy for Huntington's disease in recent years. Utilizing AAV2 vectors carrying CRISPR/Cas9 technology-related components targeting mHTT can modify the alleles where HTT mutations occur, removing trinucleotide CAG repeat expansion and restoring normal expression (Yan et al., 2023; Shin et al., 2016). Accordingly, this technology has also been developed for ASOs targeting mHTT to inhibit mHTT expression (Southwell et al., 2014). Using high-throughput technology to screen proteins that inhibit the cytotoxicity of mHTT proteins and combining them with gene therapy technology can also provide new ideas for the treatment of diseases. The specific ideas are as follows: First, genome-wide screening technology is used to screen proteins that can promote the degradation of mHTT protein or inhibit its formation, and subsequently, the AAV vector is used to carry the cDNA of these proteins into the pathogenic tissues and express them to remove the mHTT protein. Using the above idea, the protein Mtf1 was successfully screened, and the cDNA of Mtf1 was successfully expressed in the brain and inhibited the formation of mHTT protein aggregates using AAV-PHP. eB vector (Ferlazzo et al., 2023). As gene therapy for HD treatment has become increasingly popular, Nanoparticles (NPs) applications have become increasingly promising. Solid lipid NPs containing curcumin (C-SLNs) is able to improve the mitochondrial function, reduce the oxidative stress and ameliorate neurobehavioral deficits in HD rat models, suggesting that C-SLNs might be a promising therapy for HD (Sandhir et al., 2014). In addition, SNPs are present in HD patients. Therefore, it is important to accurately determine the patient's SNPs during the diagnosis process and then design appropriate gene therapy ideas to silence the expression of the mHTT protein (Fields et al., 2021).

## 7 Gene therapy in ALS

Amyotrophic lateral sclerosis (ALS) is a fatal motor neuron degenerative disease characterized by the loss of motor neurons. It is usually characterized by progressive atrophy of the muscles of the limbs, trunk, chest, and abdomen, leading to muscle weakness and rigidity, and ultimately death as a result of respiratory failure 3–5 years after onset (Niedermeyer et al., 2019). Currently, gene therapy for ALS primarily focuses on SOD1 mutations, C9orf72 hexanucleotide repeat expansions, ATXN2 trinucleotide expansions, FUS mutations and sporadic disorder without known genetic component. Recent clinical advancements have highlighted targeted delivery strategies for gene regulation, including ASOs, RNAi and CRISPR. These strategies particularly emphasize the mutated SOD1 and C9orf72 genes (Kim et al., 2020; Amado and Davidson, 2021).

It has been found that mutations in superoxide dismutase (SOD1) account for ~10 to 20 percent of the incidence of familial inherited ALS (fALS; Wong et al., 1995). Subsequent studies confirm these findings (Chia et al., 2018). Mutations in the SOD1 lead to noticeable alterations in protein conformation, followed by induced protein aggregation and abnormal motor neuron function (Bruijn et al., 1998). ASOs targeting SOD1 (ASOs-SOD1) are designed to reduce SOD1 expression, which holds promise for slowing the progression of ALS and prolonging patient survival during clinical treatment (Miller et al., 2020). In addition, shRNA targeting SOD1 to be transported by AAV9 vector (AAV9-SOD1-shRNA) and CRISPR-Cas9 targeting SOD1 to be transported by AAV9 can reduce mutant SOD1 protein, thus improving motor function and prolonging survival in mouse models of ALS (Foust et al., 2013; Gaj et al., 2017). Two patients with fALS caused by SOD1 mutations received an intrathecal injection with AAV vectors encoding miRNAs targeting SOD1. one of the patients was noted to have stable ALS Functional Rating Score, the other displayed slight improvement in right leg strength (Mueller et al., 2020). These findings suggest that SOD1 may be a potential target for ALS therapy.

The occurrence of GGGGCC hexanucleotide repeats (G4C2 hexanucleotide repeat expansion, HRE) in intron 1 of the noncoding region of C9ORF72 accounts for ~23.5 percent of the total incidence of fALS (DeJesus-Hernandez et al., 2011). HRE leads to aberrant expression of C9ORF72 and can translate neurotoxic dipeptide repeat proteins (DPRs) depending on the non-AUG mechanism (Haeusler et al., 2016). In addition, the accumulation of HRE-encoded sense and antisense transcripts in motor neurons can form RNA foci (Haeusler et al., 2016). ASOs targeting the maturation of C9ORF72 pre-mRNA processing (ASOs-C9ORF72) have been designed to effectively reduce the level of RNA foci and improve disease symptoms (Jiang et al., 2016).

TAR DNA-binding protein-43 (TDP-43) encoded by TARDBP is the most extensively studied and best characterized in the ALS. Almost all of patients with ALS develop cytoplasmic inclusions of the TDP-43 protein. However, mutations in TARDBP account for < 4% of fALS cases and < 1% of sALS (Zou et al., 2017; Giordana et al., 2010). TDP-43 regulates alternative splicing of stathmin-2 (STMN2) pre-mRNA, which in turn regulates protein expression. STMN2 controls neuronal damage repair and regeneration. Abnormal cytoplasmic aggregation of TDP-43 protein fails to translate exon 2a-(exon-2a) into STMN2 mRNA, which in turn leads to truncation of the STMN2 protein, forming polyadenylated STMN2 protein, ultimately causing the death of motor neurons (Chauvin and Sobel, 2015). Nemo-like kinase (nlk) inhibits the biogenesis of lysosomes, and ASOs targeting silencing nlk (ASOs-nlk) are designed to effectively promote the formation of lysosomes, followed by degradation of TDP-43 protein aggregates in the cytoplasm, leading to normal expression of STMN2 (Tejwani et al., 2023). ASOs targeting STMN2 can also be designed to restore the expression of exon-2a in STMN2 mRNA, leading to normal STMN2 expression (Baughn et al., 2023). Along these lines, TDP-43 and STMN2 can be targeted to develop appropriate gene therapy strategies. for future gene therapies for ALS. To achieve precise treatment, gene therapy should accurately analyze disease-causing gene mutations in patients with ALS, which will provide new ideas for the clinical treatment of ALS.

## 8 Gene therapy in SMA

Spinal muscular atrophy (SMA) is a type of autosomal recessive NMDs. It can be divided into five types according to clinical manifestations and time of onset: SMA-0, SMA-I, SMA-II, SMA-III, and SMA-IV. SMA-0 is a congenital type characterized by thinness at birth, severe muscular and joint atrophy, facial paralysis, respiratory failure, and death at ~2 years of age. It develops from 6 to 18 months of age and is characterized clinically by loss of deep tendon reflexes, fine tremor of the upper extremities, joint contractures, kyphosis, and ventilator-assisted respiration. SMA-III develops in childhood after 18 months of age, and the patient can walk independently but gradually loses the ability to walk and develops chewing difficulties with the progression of the disease. SMA-IV is an adult-type disease, and most patients develop the disease after 35 years of age. SMA-IV is typically characterized by an insidious onset and slow progression and causes a reduction in physical functioning over time, culminating in organ failure and death (Mercuri et al., 2022). Although the clinical features of SMA are complex, its etiology is relatively simple. Ninety-five percentage of patients present with dysfunctional SMN protein caused by a homozygous deletion of the SMN1 gene exon-7 in motor neurons, resulting in the damage and death of motor neurons (Lefebvre et al., 1995). The SMN2 gene, which differs from the SMN1 gene by only one base, can also encode the SMN protein, in which pre-mRNA SMN2 removes exon-7 from the mRNA after alternative splicing, ultimately forming an incomplete and easily degradable SMN protein (Han et al., 2012). Although SMN2 encodes an unstable protein, its copy numbers within motor neurons correlate positively with the time of onset and negatively with malignancy. The number of SMN2 copies in SMA-0 and SMA-I was almost 2, SMN2 copies number in SMA-II was usually 3 SMN2 copies number in SMA-III and SMA-IV was usually greater than or equal to 4 (Nishio et al., 2023). Gene therapies approved by the Food and Drug Administration (FDA) for SMA include supplementation of the brain with the WT-SMN1 gene to express the WT-SMN protein and regulate the SMN2 gene (Ogbonmide et al., 2023).

**Figure 3:**
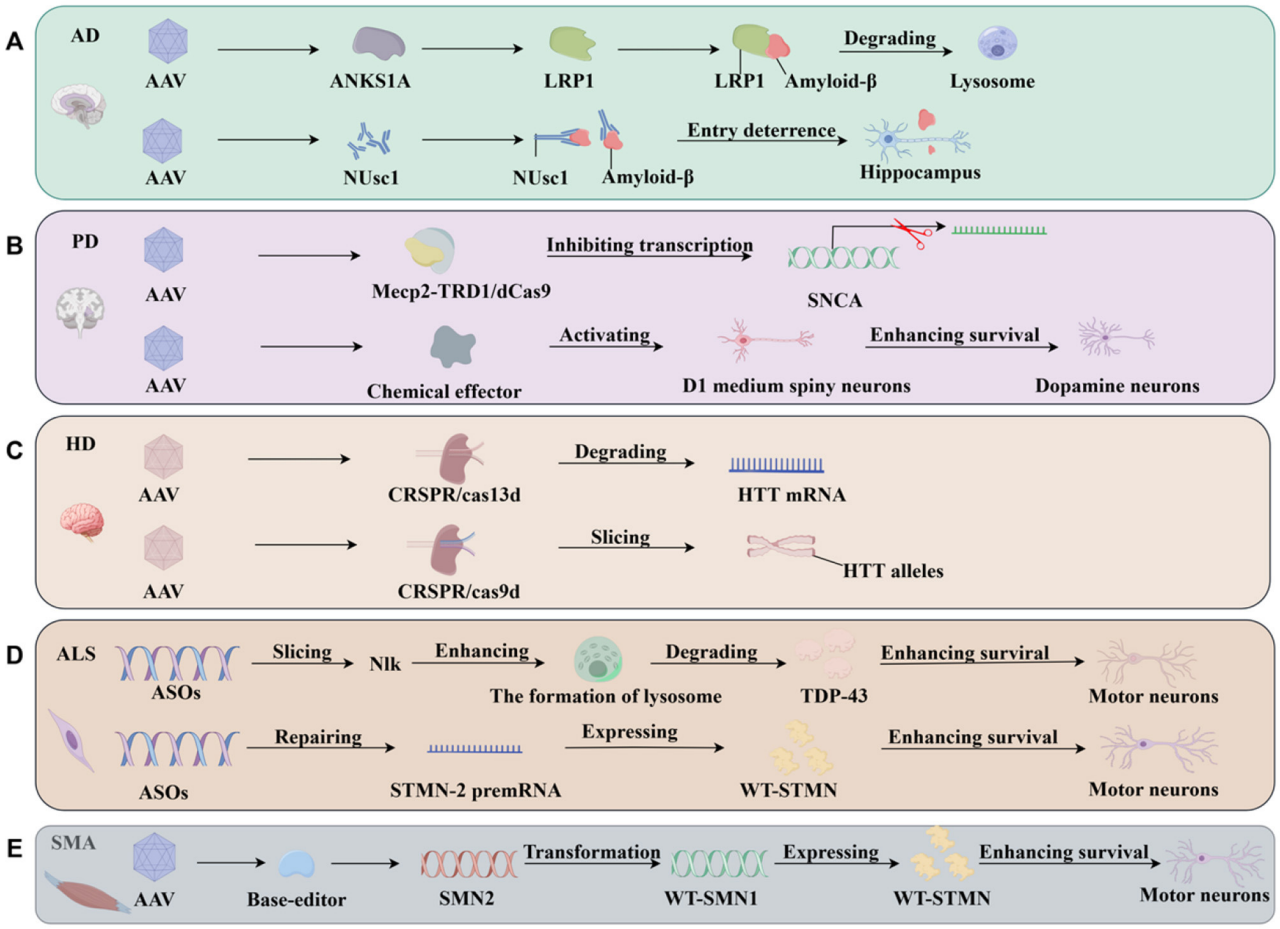
Therapeutic strategies of gene therapy targeting neurodegenerative diseases. **(A)** The role of gene therapy in AD. The expression of the ANKS1A protein can activate LRP1 and promote the formation of a complex between LRP1 and amyloid-β and transported the complex to the lysosome to remove amyloid-β. NUsc1 targeting amyloid-β can inhibit the binding of amyloid-β to neurons, and enhance the survival of hippocampus. **(B)** The role of gene therapy in PD. MeCp2-TRD binds to dCas9 to inhibit SNCA transcription, thereby repressing SNCA transcription. The chemo-genetic effector allows precise D1-MSN activation and inhibits the death of dopaminergic neurons. **(C)** The role of gene therapy in HD. miRNAs and shRNAs are used to target the silencing of HTT protein expression and reduce the number of mHTT proteins. Utilizing AAV2 vectors carrying CRISPR/Cas9 technology-related components targeting the silencing of alleles. **(D)** The role of gene therapy in ALS. ASOs targeting silencing nlk (ASOs-nlk) promote the formation of lysosomes, followed by degradation of TDP-43 protein aggregates to enhance the survival of motor neurons. ASOs targeting STMN2 can also restore the expression of STMN2 pre-mRNA, increase WT STMN expression and then enhance the survival of motor neurons. **(E)** The role of gene therapy in SMA. AAV-9 base editor achieves genetic modifications of SMA change from SMN2 to SMN1, increase the expression of the WT-SMN protein and enhance the survival of motor neurons. AD, Alzheimer's disease; AAV, adeno-associated virus; ANKS1A, ankyrin repeat and SAM domain containing 1 A; LRP1, lipoprotein receptor-related protein 1; NUsc1, an oligomer-specific single-chain variable-fragment antibody; PD, Parkinson's disease; MeCp2, methyl CpG binding protein 2; TRD, transcription repression domain; HD, Huntington's disease; HTT, Huntingtin's gene; CRISPR, clustered regularly interspaced short palindromic repeats; ASOs, antisense oligonucleotide; NLK, Nemo-like kinase; TDP43, TAR DNA-binding protein-43; STMN2, stathmin-2.

In the clinical treatment approved by the FDA in 2019, AAV-9 (AVXS-101) with WT-SMN1 cDNA by intravenous injection in neonatal patients with SMA-0 and SMA-I was utilized to effectively improve the function of motor neurons and enhance the ability of patients to walk and swallow independently, ultimately prolonging survival (Mendell et al., 2017; Al-Zaidy et al., 2019). In another FDA-approved clinical trial, ASOs were designed for neonates and infants with SMA-0, SMA-I, and SMA-II via intrathecal injection to regulate the pre-mRNA alternative splicing of SMN2 (ASOs-SMN2 mRNA) to preserve exon-7 at the mRNA level and achieve WT-SMN protein expression, which can effectively improve patient symptoms and enhance patient survival (Passini et al., 2011; Darras et al., 2019; Finkel et al., 2017, 2016). Moreover, in FDA-approved clinical trials, the intake of small molecules, including risdiplam and branaplam, can regulate alternative splicing of SMN2 pre-mRNA via oral administration, which also holds promise for application in clinical treatment and surpasses the therapeutic effects of the aforementioned therapies (Naryshkin et al., 2014; Cheung et al., 2018). A series of small molecules has also been screened using high-throughput omics to restore proper expression of the normal WT-SMN protein, which can also provide new ideas for the combination of SMA gene therapy in subsequent trials (Ottesen et al., 2023). In addition, the AAV9 vector targeting nucleases or a base editor (AAV-9 base editor) achieves genetic modifications of SMA with single-base pair precision, causing it to change from SMN2 to SMN1 and increase the expression of the WT-SMN protein (Arbab et al., 2023). Similarly, the construction of AAV delivery vectors with CRISPR/Cas technology elements into motor neurons shifts SMN2 to SMN1 by homology-dependent repair (HDR), promoting the expression of WT-SMN proteins (Zhou et al., 2018), both of which could be developed into new clinical therapeutic options in the future. In future clinical treatment of SMA, gene therapy strategies need to be developed for application in the treatment of patients with SMA-III and SMA-IV, as well as for an accurate safety assessment and prognostic analysis of existing clinical treatment strategies. The main findings that emerged in clinical trials of genetic therapies for NDDS are briefly summarized as follows Table 2. We also summarized the role of gene therapy in AD, PD, HD ALS, SMA in Figure 3.

**Table 2:** Gene therapy trials for neurodegenerative diseases.

**Disease**	**Gene therapy**	**Delivery route**	**Phase**	**Trial code**
AD	AAV2-NGF	Intraparenchymal injection	Phase II	NCT00876863
			Phase I	NCT00087789
	ASOs-MAPT	Intravenous injection.	Phase II	NCT03186989
	AAVrh.10-hAOPE2	Intraparenchymal injection	Phase I	NCT03634007
	AAV2-hTERT	Intravenous-spinal intrathecal dual injection	Phase I	NCT04133454
HD	ASOs-total HTT	Intrathecal injection	Phase III	NCT02519036
			Phase II	NCT03342053
			Phase I	NCT04000594
			Phase III	NCT03761849
			Phase III	NCT03842969
	AAV5-miHTT	Intraparenchymal injection	Phase II	NCT04120493
	ASOs-HTT mutant	Intrathecal injection	Phase II	NCT03225833
			Phase II	NCT03225846
PD	AAV2-AADC	Intraparenchymal injection	Phase II	NCT02418598
			Phase I	NCT00229736
			Phase I	NCT01973543
			Phase I	NCT03065192
			Phase II	NCT03562494
	AAV2-GDNF	Intraparenchymal injection	Phase I	NCT01621581
			Phase I	NCT04167540
	AAV2-neurturin		Phase II	NCT00400634
	AAV2-GAD	Intraparenchymal injection	Phase II	NCT00643890
			Phase II	NCT00195143
	AAV9-GBA1	Intracisternal injection	Phase II	NCT04127578
			Phase I	NCT02906020
	AAV2-NTN	Intraparenchymal injection	Phase I	NCT00252850
			Phase II	NCT00400634
			Phase II	NCT00985517
	ASOs-LRRK2	Intraparenchymal injection	Phase I	NCT03976349
ALS	ASOs-SOD1	Intrathecal injection	Phase I	NCT01041222
			Phase I	NCT03764488
			Phase II	NCT02623699
			Phase III	NCT03070119
	ASOs-C9ORF172	Intrathecal injection	Phase I	NCT03626012
			Phase I	NCT04288856
SMA	ASO-SMN2	Intrathecal injection	Phase I	NCT01494701
			Phase I	NCT01780246
			Phase I	NCT02052791
			Phase I	NCT04050852
			Phase II	NCT01703988
			Phase II	NCT01839656
			Phase II	NCT02386553
			Phase III	NCT02292537
			Phase III	NCT02594124
			Phase III	NCT04089566
	AAV9-SMN1	Intravenous injection	Phase I	NCT02122952
			Phase I	NCT03381729
			Phase III	NCT03306277
			Phase III	NCT03461289
			Phase III	NCT03505099
			Phase III	NCT03837184

## 9 Other therapeutic target of gene therapy targeting NDDs

The formation of protein aggregates in neurons is a common pathogenesis of NDDS, and activation of internal physiological effects in neurons can also remove protein aggregates. The development of gene therapy strategies has potential for clinical applications.

Currently, the following therapeutic targets are in preclinical stages of investigation and may be applied to the clinical treatment of NDDS patients in the future: abnormal activation of the mTOR signaling pathway is positively correlated with the accumulation of pathology-like proteins in the neurons, and therapeutic strategies to inhibit the activation of the mTOR signaling pathway can be developed for the treatment of NDDS (Hoeffer and Klann, 2010; Stoica et al., 2011; Laplante and Sabatini, 2012). Protein aggregates localized to the lumen of the endoplasmic reticulum (ER) lead to an imbalance in ER calcium homeostasis, which in turn induces endoplasmic reticulum stress (ER stress; Cabral-Miranda and Hetz, 2018). ER stress activates the UPR signaling pathway to maintain a proper balance in intracellular protein homeostasis. However, the activated UPR signaling pathway promotes neuronal apoptosis and new treatment strategies can inhibit the activation of the UPR signaling pathway (Xu et al., 2023). Mitochondrial dysfunction is also positively associated with the progression of neurodegenerative diseases, and regulatory molecules in gene therapy may promote mitochondrial biogenesis or inhibit mitochondrial oxidative stress, thereby promoting the clearance of protein aggregates (Chiang et al., 2018). Epigenetic modifications, such as DNA methylation, chromatin remodeling, and histone post-translational modifications, are highly correlated with the formation of protein aggregates and for epigenetic modification of pathological genes or proteins, gene therapy strategies targeting the silencing or expression of epigenetic modification-associated enzymes are utilized to modulate the expression of pathologically relevant mutant genes and pathological protein activity. Gene therapy strategies for silencing or expressing epigenetic modification-related enzymes can achieve the purpose of regulate the expression of pathology-related mutated genes and pathological protein activities (Berson et al., 2018). Autophagy is an evolutionary ancient and highly conserved physiological process by which cells degrade dysfunctional intracellular organelles and denatured proteins. Gene therapy can be used to modulate the expression of autophagy-related molecules to accelerate the formation of autophagosomes and remove protein aggregates (Miller and Thorburn, 2021). Based on the above ideas, the gene therapy strategies developed for NDDS in recent years are summarized as follows Table 3.

**Table 3:** Other therapeutic targets of gene therapy in NDDs.

**Target**	**Disease**	**Gene therapy**	**References**
UPR signaling pathway	PD	AAV-Bip	(Gorbatyuk and Gorbatyuk, 2013)
	HD	AAV-XBP1	(Zuleta et al., 2012)
	AD	AAV-XBP1	(Duran-Aniotz et al., 2023)
mTOR signaling pathway	HD	AAV-caRheb	(Wu et al., 2017)
Mitochondrial function	PD	AAV-HSP70	(Hu et al., 2025)
Epigenetic modifications	AD	AAV-PINK1	(Wang et al., 2022)
Autophagy	PD	AAV-LAMP2A	(Issa et al., 2018)
	PD	AAV-TFEB	(Arotcarena et al., 2019)

## 10 Conclusions

In recent decades, an increasing number of preclinical and clinical studies have focused on the use of gene therapy for the prevention and treatment of neurodegenerative diseases (NDDs). Although the pervasive affliction of NDDs was deemed resistant to gene therapy, progress in vector technologies now facilitates the broad dissemination of genes into the central nervous system (CNS). The use of vectors in combination with gene editing tools that have been developed so far has the potential to replace existing clinical drug regimens. Alzheimer's disease (AD) is the most common neurodegenerative disorder. Large-scale data analysis from genome-wide association studies (GWAS) indicates that apolipoprotein E (apoE) plays a key role in AD pathogenesis. However, no clinical therapy targeting apoE is currently available for treating AD. The formation of Aβ plaques and the accumulation of tau into neurofibrillary tangles are pathological consequences of Alzheimer's disease. Current approaches to gene therapy for patients with AD are based on promoting the degradation of tau and removing amyloid aggregation (Huang and Mahley, 2014; Zhao et al., 2018). These studies are limited by the extensive amyloid pathology in AD. There is an urgent need to understand how apoE contributes to AD and to translate this knowledge into sustainable, meaningful clinical treatments. Parkinson's disease (PD) is a multifaceted disease with many contributing genes. There is evidence suggesting that the products of SNCA, LRRK2, and GBA1 may be involved in the pathogenesis of PD and may be relevant for disease progression (Nalls et al., 2019). Therefore, targeting the expression of pathogenic mutations is a promising strategy for designing disease-modifying therapies in PD. Corresponding treatment strategies can be applied for treating PD by targeted silencing of the disease-causing genes or promoting the expression of genes that can improve the survival of dopaminergic neurons. Huntington's disease (HD) is caused by an expansion of a CAG repeat within the HD gene, encoding an expanded stretch of polyglutamines in the mutant huntingtin (mHTT) protein. Many genome editing technologies have been evaluated to decrease mHTT aggregate formation and reduce mHTT-induced neurotoxicity by decreasing mHTT expression at the gene, mRNA, or protein levels (Fields et al., 2021; Sun et al., 2016). Therefore, correcting the mHTT genetic mutation through gene-editing technology may represent a powerful therapeutic approach for HD. Amyotrophic lateral sclerosis (ALS) is a chronic, progressive, and currently untreatable neurodegenerative disease. A paucity of therapeutic targets and the difficulty of accessing the relevant regions of the CNS pose challenges for gene therapy in ALS. SOD1 mutations, C9ORF72 hexanucleotide repeat expansions, and TARDBP mutations are potential therapeutic targets for gene therapy, which can be administered to reach some of the body's most protected regions, such as the cerebral cortex and the anterior horn cells of the spinal cord. Once administered to their targets, these therapies can operate through knockdown-mediated alleviation of gain-of-function mechanisms or through the introduction of protective agents, leveraging the immense potential of gene therapy. Spinal muscular atrophy (SMA) is primarily caused by deletions or homozygous mutations in the survival motor neuron 1 (SMN1) gene. The ability to provide targeted, sustained treatment gives gene therapy the potential to permanently alter the therapeutic landscape for SMA, especially in SMA-0, SMA-I, and SMA-II. Patients with favorable clinical outcomes provide an optimistic outlook for the design of future clinical trials in SMA-III and SMA-IV. More studies are required to enable a greater number of SMA patients to achieve excellent symptom control and improved quality of life.

However, there are still pressing issues that need to be addressed, such as the design of more efficient delivery vectors, off-target effects of gene editing tools, design of more efficient delivery routes, selection of therapeutic targets, and a growing understanding of the pathogenesis and progression of NDDs. Notably, precise gene editing is essential for gene therapy. Gene editing has limited efficacy, including delivery efficiency, delivery efficiency, long-term safety, immune responses and cost. Solving these problems will provide new ideas for the application of gene therapy in the clinical treatment of neurodegenerative diseases and provide hope for precise, differentiated, and individualized diagnosis and treatment.
